# A mark of disease: how mRNA modifications shape genetic and acquired pathologies

**DOI:** 10.1261/rna.077271.120

**Published:** 2021-04

**Authors:** Eliana Destefanis, Gülben Avşar, Paula Groza, Antonia Romitelli, Serena Torrini, Pınar Pir, Silvestro G. Conticello, Francesca Aguilo, Erik Dassi

**Affiliations:** 1 Department of Cellular, Computational and Integrative Biology (CIBIO), University of Trento, 38123 Trento, Italy; 2 The EPITRAN COST Action Consortium, COST Action CA16120; 3 Department of Bioengineering, Gebze Technical University, 41400 Kocaeli, Turkey; 4 Department of Medical Biosciences, Umeå University, 901 87 Umeå, Sweden; 5 Wallenberg Center for Molecular Medicine, Umeå University, 901 87 Umeå, Sweden; 6 Core Research Laboratory, ISPRO—Institute for Cancer Research, Prevention and Clinical Network, 50139 Firenze, Italy; 7 Department of Medical Biotechnologies, Università di Siena, 53100 Siena, Italy; 8 Institute of Clinical Physiology, National Research Council, 56124 Pisa, Italy

**Keywords:** RNA modifications, epitranscriptomics, mRNA, posttranscriptional regulation of gene expression, human disease, cancer

## Abstract

RNA modifications have recently emerged as a widespread and complex facet of gene expression regulation. Counting more than 170 distinct chemical modifications with far-reaching implications for RNA fate, they are collectively referred to as the epitranscriptome. These modifications can occur in all RNA species, including messenger RNAs (mRNAs) and noncoding RNAs (ncRNAs). In mRNAs the deposition, removal, and recognition of chemical marks by writers, erasers and readers influence their structure, localization, stability, and translation. In turn, this modulates key molecular and cellular processes such as RNA metabolism, cell cycle, apoptosis, and others. Unsurprisingly, given their relevance for cellular and organismal functions, alterations of epitranscriptomic marks have been observed in a broad range of human diseases, including cancer, neurological and metabolic disorders. Here, we will review the major types of mRNA modifications and editing processes in conjunction with the enzymes involved in their metabolism and describe their impact on human diseases. We present the current knowledge in an updated catalog. We will also discuss the emerging evidence on the crosstalk of epitranscriptomic marks and what this interplay could imply for the dynamics of mRNA modifications. Understanding how this complex regulatory layer can affect the course of human pathologies will ultimately lead to its exploitation toward novel epitranscriptomic therapeutic strategies.

## INTRODUCTION

RNA molecules can undergo more than 170 different chemical modifications (Boccaletto et al. 2018). These marks can decorate many types of RNA species, both coding and noncoding RNA (ncRNA), including messenger RNA (mRNA), ribosomal RNA (rRNA), transfer RNA (tRNA) and others. This ever-expanding set of RNA modifications, collectively referred to as the epitranscriptome, has recently emerged as a widespread facet of cotranscriptional and posttranscriptional gene expression regulation (Laurencikiene et al. 2006; Saletore et al. 2012; Nachtergaele and He 2017; Roundtree et al. 2017; Martinez and Gilbert 2018; Zhao et al. 2018). These regulatory layers are key determinants of protein levels and cellular phenotypes (Halbeisen et al. 2008; Vogel et al. 2010; Schwanhäusser et al. 2011; Corbett 2018).

A broad set of RNA-binding proteins (RBPs) determines the mRNA epitranscriptome: Modifications are induced by writers, and several can be reverted by erasers. Eventually, some modifications need readers to be decoded. (Kadumuri and Janga 2018; Nachtergaele and He 2018; Delaunay and Frye 2019; Quinones-Valdez et al. 2019). Through the action of these RBPs, the epitranscriptome controls processes ranging from alternative splicing and polyadenylation to RNA stability, localization, and translation (Gerstberger et al. 2014; Bartel 2018). These regulators form complex networks of interaction leading to a dynamic control of gene expression with deep implications for cellular physiology and pathology (Wurth and Gebauer 2015; Dassi 2017; Quattrone and Dassi 2019; Zanzoni et al. 2019). Given their relevance in multiple cellular functions, alterations of RNA modifications and their modifying enzymes have been observed in a broad range of human diseases, including cancer, neurological disorders and several others (Meier et al. 2016; Jonkhout et al. 2017; Angelova et al. 2018; Jain et al. 2018; Christofi and Zaravinos 2019; Huang et al. 2020b).

**FIGURE 1.**
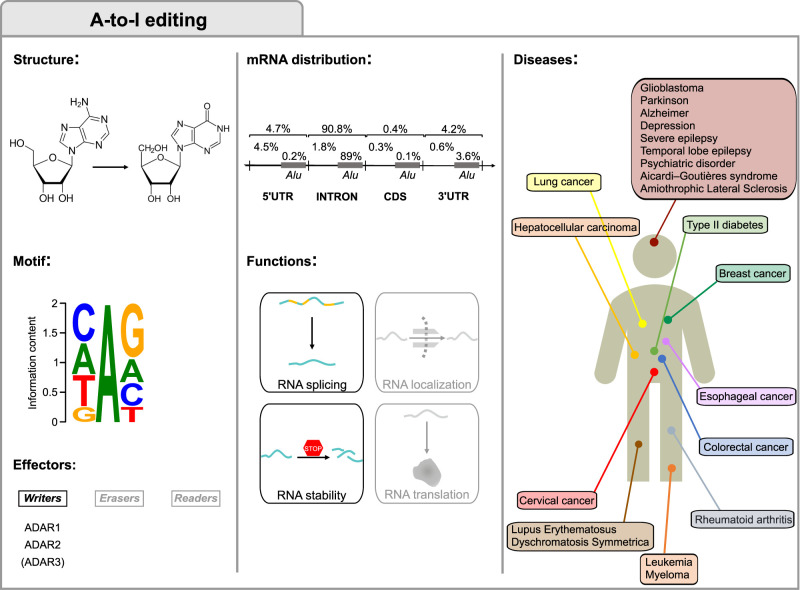
A-to-I editing. The first column displays the structures of adenosine and inosine involved in the deamination, the consensus motif and the A-to-I editing main effectors. The motif was obtained by data in Cohen-Fultheim and Levanon (2021) and plotted with WebLogo (Crooks et al. 2004). The central column shows the percentage of editing at nonrepetitive regions and Alu repeats and the functions in mRNA fate. The third column displays A-to-I editing-associated disorders and the organs to which they are associated.

**FIGURE 2.**
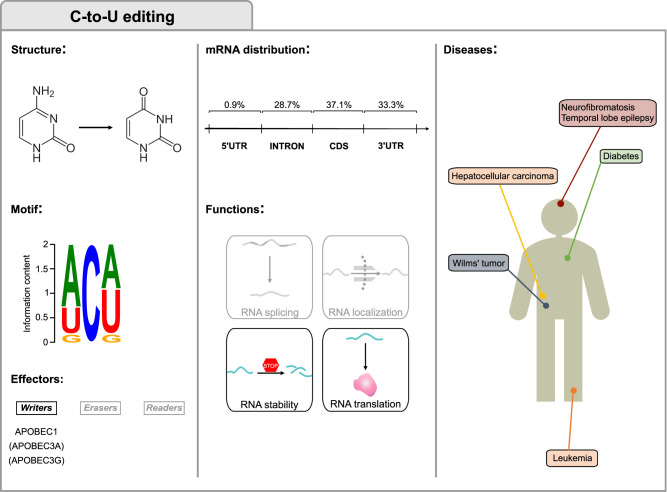
C-to-U editing. The first column displays the structures of cytidine and uridine involved in the deamination, the consensus motif and the C-to-U editing main effectors. The motif was obtained by data in Rosenberg et al. (2011) and plotted with WebLogo (Crooks et al. 2004). The central column shows the percentage of editing in the mRNA regions and the functions in mRNA fate. The third column displays C-to-U editing-associated disorders and the organs to which they are associated. Considering that little is known on the significance of RNA editing by APOBEC3A and APOBEC3G, all features in the figure relate to APOBEC1, and APOBEC3A/APOBEC3G are only mentioned in parentheses.

**FIGURE 3.**
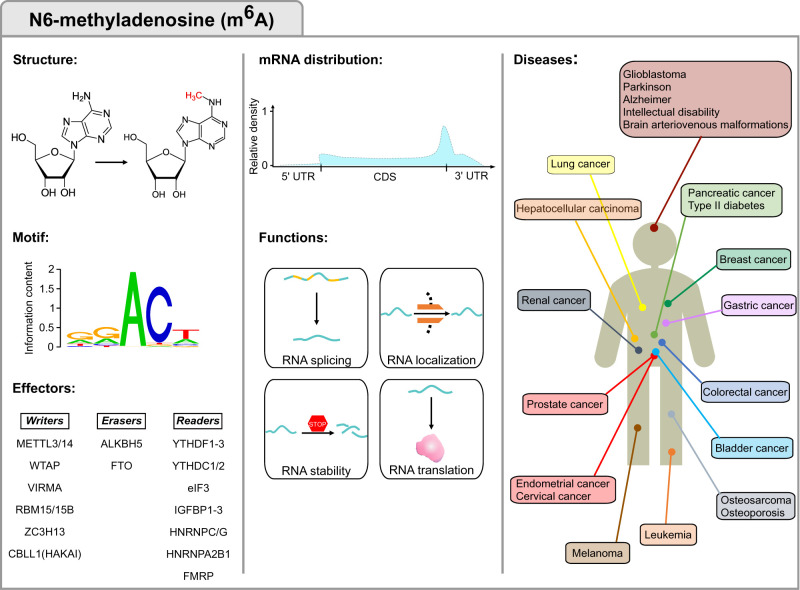
N^6^-methyladenosine (m^6^A) modification. The first column displays the m^6^A structure, consensus motif and m^6^A machinery factors. The motif was obtained by data in Linder et al. (2015) and plotted with WebLogo (Crooks et al. 2004). The central column highlights the m^6^A distribution and functions in mRNA fate, while the third column displays m^6^A-associated disorders and the organs to which they are associated.

**FIGURE 4.**
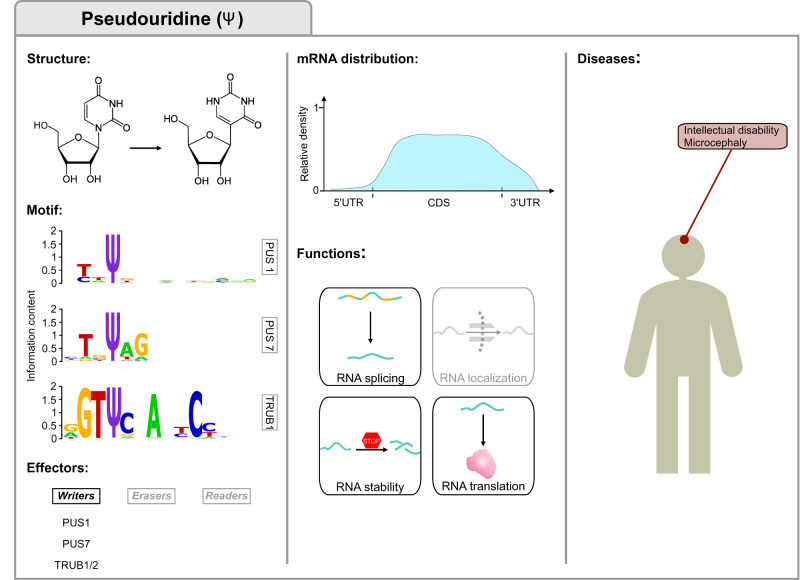
Pseudouridine (Ψ) modification. The first column displays the Ψ structure, consensus motifs and lists the main writers of Ψ in mRNAs. The motif was obtained by data in Carlile et al. (2019) and plotted with WebLogo (Crooks et al. 2004). The central column highlights Ψ distribution (Carlile et al. 2019) and functions in mRNA fate. The third column displays the disorders associated with dysregulated pseudouridylation.

In this review, we will describe mRNA modifications and their increasingly appreciated role as drivers of human pathologies. We will give particular focus on the most abundant ones, namely RNA editing (A-to-I and C-to-U), *N*^6^-methyladenosine (m^6^A), and pseudouridine (Ψ), for which we provide flashcards (Figs. 1–4) summarizing their most important features and disease associations, and a comprehensive list of disease-related modified sites (Supplemental Table S1). Furthermore, we will provide an overview of detection methods and discuss emerging evidence on the interplay of different modifications, proposing potential avenues to improve our understanding of these pervasive RNA regulators.

## EPITRANSCRIPTOMIC MARKS

### RNA editing by deamination

RNA editing, mediated by several enzymes belonging to a zinc-binding superfamily of deaminases, targets most types of cellular RNAs. A-to-I is the most common form of editing in human cells and is performed by the adenosine deaminase acting on RNA (ADAR) enzymes (Bass 2002; Mannion et al. 2015; Eisenberg and Levanon 2018). Alongside A-to-I editing, C-to-U editing is performed by the Apolipoprotein B mRNA editing enzyme, catalytic polypeptide-like (APOBEC) family of enzymes. Both *ADAR* and *APOBEC* gene families likely originate from the adenosine deaminase acting on tRNA (*ADAT*) genes (Gerber and Keller 2001; Conticello et al. 2007), whose encoded proteins edit the wobble position of many tRNAs (Torres et al. 2014).

#### A-to-I editing

In humans, A-to-I editing (Fig. 1) is mediated by ADAR1 and ADAR2, while the catalytically inactive ADAR3 can modulate the process. These enzymes act as homodimers and deaminate adenosines within double-stranded regions of RNA (Gallo et al. 2003; Thuy-Boun et al. 2020). Binding to the target region is mediated by double-stranded RNA (dsRNA) binding domains. Since inosines that result from editing are read as guanosines by the translational machinery, editing can recode the mRNA and lead to the translation of proteins different from those specified by the genome, thus increasing the complexity of the transcriptome.

The first edited sites were discovered on the transcripts of the glutamate receptor 2 (*GRIA2*) and the serotonin 5-hydroxytryptamine (2C) (*5-HT_2c_*) receptors (Sommer et al. 1991; Higuchi et al. 1993; Burns et al. 1997). *GRIA2* editing is essential for brain development as it allows formation of heteromeric complexes modulating neuronal function. Historically, the main role of A-to-I editing was considered to be recoding, mainly due to the importance of ADAR2-mediated editing in brain development (Brusa et al. 1995; Higuchi et al. 2000). However, it soon became evident that many edited sites lie outside the coding regions (Athanasiadis et al. 2004; Kim et al. 2004; Levanon et al. 2004; Li et al. 2009; Bazak et al. 2014; Picardi et al. 2016, 2017; Eisenberg and Levanon 2018). Most A-to-I RNA editing sites occur on noncoding sequences such as 5′ and 3′ untranslated regions (UTRs) (Chen and Carmichael 2012), introns, and microRNAs (miRNAs) (Luciano et al. 2004; Blow et al. 2006; Yang et al. 2006). In humans, most of these sites lie in *Alu* sequences, ancient retrotransposons whose repeated sequences facilitate formation of double-stranded structures (Athanasiadis et al. 2004; Kim et al. 2004; Levanon et al. 2004). ADAR-mediated editing of noncoding regions can modulate the RNA fate and function. For example, changes in their primary sequence can affect how they are targeted by miRNAs (Roberts et al. 2018) or alter transcript splicing (Rueter et al. 1999). More importantly, insertion of I:U mismatches in place of A:U pairs can alter the structure of the RNA itself, affecting transcript interactions and stability (Wang et al. 2013). Indeed, ADAR1 deficiency leads to accumulation of cytoplasmic dsRNA that, being interpreted as a sign of viral infection, leads to the activation of the cellular response to dsRNA through RIG-I and MDA5 (Mannion et al. 2014; Liddicoat et al. 2015; Pestal et al. 2015). ADAR1 homozygous deficiency in mice induces embryonic lethality (Wang et al. 2000).

ADAR1 also plays a role in the physiological interferon-mediated cellular response, as widespread editing prevents translational shutdown and cell death (Hartner et al. 2009; Chung et al. 2018). Missense mutations in *ADAR1* cause Aicardi–Goutières Syndrome, a childhood autoimmune encephalitis characterized by increased interferon (Rice et al. 2012; Gallo et al. 2017). Mutations in *ADAR1* are also associated with dyschromatosis symmetrica hereditaria (DSH), a rare autosomal genetic disorder of the skin, but the pathogenetic mechanisms are not yet clear (Miyamura et al. 2003; Kono and Akiyama 2019).

Deficiencies of A-to-I RNA editing mediated by ADAR2 have instead been associated with diseases of the central nervous system (Costa Cruz and Kawahara 2021). Increased levels of *GRIA2* editing have been found in epileptic patients (Vollmar et al. 2004). In amyotrophic lateral sclerosis (ALS), alterations in editing levels of *GRIA2* and other transcripts may contribute to the disease (Kawahara et al. 2004; Kwak et al. 2008; Donnelly et al. 2014). Similarly, decreases in editing levels of the *5-HT2C* serotonin receptor affect serotonin production and are involved in several psychiatric disorders (Sodhi et al. 2001; Grohmann et al. 2010; O'Neil and Emeson 2012; Weissmann et al. 2016), and it has also been found in the prefrontal cortex of suicide victims (Gurevich et al. 2002a,b). Reduced editing was also observed in Alzheimer's patients (Khermesh et al. 2016; Franzén et al. 2018). Moreover, probably due to their involvement in interferon response, ADAR enzymes may play a role in autoimmune diseases, such as lupus erythematosus (Laxminarayana et al. 2002, 2007; Orlowski et al. 2008; Vlachogiannis et al. 2020).

Alterations in A-to-I editing have also been associated with cancer (Kung et al. 2018). On one hand, hypo-editing in *Alu* repeats has been observed in several tumor types (Paz et al. 2007). Low levels of *GRIA2* editing were observed in human gliomas (Maas et al. 2001) and overall editing levels have been used to stratify glioblastoma patients (Tomaselli et al. 2015; Silvestris et al. 2019). On the other hand, increased levels of ADAR1 have been observed in esophageal, lung carcinomas (Qin et al. 2014; Anadón et al. 2016) in lymphoproliferative diseases (Beghini et al. 2000; Jiang et al. 2013; Lazzari et al. 2017) and in hepatocellular carcinoma (Chen et al. 2013), sometimes associated with poor prognosis. Editing of *AZIN1* is correlated to hepatocellular carcinoma and it is involved in cell proliferation and invasion by maintaining polyamine homeostasis (Chen et al. 2013; Qin et al. 2014; Shigeyasu et al. 2018) and high levels of A-to-I editing of the Ras homolog family member Q increase tumor invasion in colorectal cancer (Han et al. 2014). Intriguingly, editing targets of ADAR2 with opposite effects have been identified in esophageal squamous cell carcinoma (Chen et al. 2017; Fu et al. 2017). Composite effects have also been observed as up-regulation of ADAR1 and down-regulation of ADAR2 promote hepatocellular carcinoma (Chan et al. 2014).

Finally, viruses can hijack the A-to-I editing machinery to trigger a proviral phenotype through editing of viral transcripts (Phuphuakrat et al. 2008; Doria et al. 2009) or cellular transcripts that modulate the cellular response (Gélinas et al. 2011; Pfaller et al. 2011; Samuel 2012).

#### C-to-U editing

C-to-U RNA editing (Fig. 2) was the first form of editing described in humans when a discrepancy between transcript and genetic sequence of the Apolipoprotein B (*APOB*) mRNA was identified in the small intestine (Chen et al. 1987). Recoding of the *APOB* transcript leads to the translation of a truncated form -ApoB48-, that allows synthesis of chylomicrons (Lo and Coschigano 2020).

Soon after, a deaminase -APOBEC1- was identified as the enzymatic core of the editing complex (Navaratnam et al. 1993; Teng et al. 1993; Blanc and Davidson 2010). APOBEC1 is a member of the AID/APOBEC family of deaminases that target cytosines in the context of single-stranded nucleic acids (Salter et al. 2016).

Contrary to ADARs, whose targeting presents only a slight sequence preference (guanines 5′ to the edited sites are not favored—e.g., Cohen-Fultheim and Levanon 2021), APOBEC1 targets nucleic acids within a stronger sequence context (adenine/uracil 5′ to the edited site, A/U rich regions—e.g., Rosenberg et al. 2011; Blanc et al. 2014). While APOBEC1 can target RNA autonomously, the editing specificity and efficiency are largely determined by its cofactors APOBEC1 complementation factor (A1CF) (Lellek et al. 2000; Mehta et al. 2000) and RNA-binding-motif-protein-47 (RBM47) (Fossat et al. 2014; Fossat and Tam 2014). Until advent of high-throughput sequencing, only a few APOBEC1-edited transcripts had been identified beyond *APOB* (Skuse et al. 1996; Yamanaka et al. 1997; Meier et al. 2005). The availability of APOBEC1 deficient murine models allowed the identification of several other editing targets of APOBEC1 in mice (Rosenberg et al. 2011; Blanc et al. 2014; Harjanto et al. 2016; Cole et al. 2017; Rayon-Estrada et al. 2017). Most of these editing sites lie in the 3′UTRs and at this time it is still difficult to envision a global role for APOBEC1 editing beyond its effect on selected specific transcripts. For example, APOBEC1 deficiency in mice promotes a proinflammatory environment in the brain, likely mediated by lack of editing in microglia, which is correlated to progressive central nervous system pathophysiology (Cole et al. 2017). A fascinating hypothesis posits that APOBEC1-mediated RNA editing might increase variability among cellular subpopulations (Harjanto et al. 2016). More important, APOBEC1-mediated RNA editing could also act as a restriction factor against viruses and mobile elements (Bishop et al. 2004; Petit et al. 2009; Ikeda et al. 2011; Di Giorgio et al. 2020). Independently from its editing activity, APOBEC1 binding to RNA could regulate transcript stability (Anant and Davidson 2000; Prohaska et al. 2014).

A potential role in disease for APOBEC1 has been envisioned since its discovery: Overexpression of APOBEC1 in the liver of several animal models induces cancer (Yamanaka et al. 1995) and its deficiency in cancer-prone mice reduces the onset of neoplastic lesions (Blanc et al. 2007). Indeed, recoding of the tumor-suppressor *NF1* (Skuse et al. 1996; Mukhopadhyay et al. 2002) leads to its inhibition, and editing of *NAT1* (Yamanaka et al. 1997) could deregulate p21 (Kung et al. 2018). Noteworthy, APOBEC1 can also target DNA and therefore, its oncogenic potential could also be derived from its mutagenic activity (Saraconi et al. 2014).

APOBEC1 might also be involved in the progression of temporal lobe epilepsy through editing of glycine receptors (Meier et al. 2005; Kankowski et al. 2018).

Considering how central *APOB* editing is for cholesterol transport in the blood—*Apobec1* deficient mice display hypercholesterolemia—mutations/polymorphisms in *Apobec1* might increase the risk of cardiovascular diseases. Yet, no inactivating mutations have been identified in humans so far.

Beyond APOBEC1, other AID/APOBECs target RNA (Sharma et al. 2015, 2016; Asaoka et al. 2019; Jalili et al. 2020). Among them, APOBEC3A is induced by hypoxia and interferon in monocytes and macrophages (Sharma et al. 2015). While its biological significance is not yet clear, increased levels of C-to-U RNA editing in tumors have been associated with improved survival, likely due to a better immune response (Asaoka et al. 2019).

### N^6^-methyladenosine

m^6^A, or methylation at the N^6^ position in adenosine (Fig. 3), is the most abundant internal modification in mRNAs and long noncoding RNAs (lncRNAs) in eukaryotes, regulating transcriptional and posttranscriptional processes that control gene expression. m^6^A was first discovered in mRNAs in 1974, and shortly after, the RRACH motif was identified as a highly conserved m^6^A consensus sequence in mammals (Desrosiers et al. 1974; Perry and Kelley 1974; Lavi and Shatkin 1975; Wei et al. 1975; Schibler et al. 1977; Wei and Moss 1977; Csepany et al. 1990; Harper et al. 1990; Linder et al. 2015). Furthermore, it has been shown that m^6^A can be added cotranscriptionally (Ke et al. 2017), and it has been predominantly located at long internal exons, near stop codons and along 3′UTRs (Dominissini et al. 2012; Meyer et al. 2012; Linder et al. 2015).

In mammalian cells, the core m^6^A methyltransferase complex, hereafter referred to as “writers,” consists of the heterodimer Methyltransferase-Like 3 (METTL3) and 14 (METTL14) (Liu et al. 2014), effecting the enzymatic activity and serving as an RNA-binding scaffold, respectively (Śledź and Jinek 2016; Wang et al. 2016b). Other writer components, reviewed elsewhere (Lence et al. 2019), are important for the deposition of m^6^A at specific transcripts. Fat mass and obesity-associated protein (FTO) and Alkb homolog 5 (ALKBH5) are the demethylases or “erasers” of m^6^A (Jia et al. 2011; Zheng et al. 2013), suggesting that this chemical modification can be formed and removed in a reversible manner. The m^6^A-mark is recognized by a group of proteins categorized as “readers” which bind and decode transcripts harboring the m^6^A modification into distinct RNA fates (Zaccara et al. 2019).

m^6^A plays a central role in several biological and pathological processes (Fig. 3; Aguilo and Walsh 2017; Malla et al. 2019). Hence, the dysregulated expression of writers, erasers, and readers, leading to aberrant m^6^A patterns, plays a role in metabolic disease, neurodegeneration, and tumorigenesis, among others. For instance, m^6^A is necessary for the function of the pancreatic β-cell, as depletion of m^6^A impairs insulin secretion by decreasing AKT phosphorylation and PDX1 protein levels (Jesus et al. 2019). Noticeably, in type 2 diabetes patients, decreased METTL3/14 expression in β cells has been observed (Jesus et al. 2019).

Furthermore, its diverse implications in neurobiological processes have been highlighted in many studies (Widagdo and Anggono 2018; Du et al. 2019; Li et al. 2019; Rockwell and Hongay 2019; Chokkalla et al. 2020). Thus, perturbations of the m^6^A machinery have been observed in numerous neuropathological states, including Alzheimer's disease, depression, and gliomas. Increased m^6^A and METTL3 levels promote the development of Alzheimer's disease (Han et al. 2020; Huang et al. 2020a). Moreover, decreased FTO expression has been correlated with increased risk of Alzheimer's disease in different ethnic populations (Reitz et al. 2012). Polymorphisms in m^6^A erasers have also been linked with increased disease risk for major depressive disorder, and attention deficit/hyperactivity disorder (Choudhry et al. 2013; Du et al. 2015; Huang et al. 2020c). However, conflicting evidence has been observed in Parkinson's disease. While a study has shown association of polymorphism in *ALKBH5* with Parkinson's disease (Qiu et al. 2020), another one, conducted on the Han Chinese population was unable to identify a significant correlation between this disease and gene variation of m^6^A players (Qin et al. 2020). Whether these antithetical results are based on ethnic differences in gene variants between the study populations or are given by gene specific m^6^A modifications remains to be clarified.

The m^6^A modification is associated with cancer. m^6^A writers, erasers, and readers act either as oncogenes or tumor suppressors in several types of cancer, although the mechanisms behind are still poorly understood (Lan et al. 2019). For instance, findings for the m^6^A machinery in breast cancer can seem controversial. Hence, high and low levels of m^6^A modification have been reported to be both oncogenic and tumor-suppressive. Recent studies have shown that METTL3 is highly expressed in breast cancer tissue compared to normal tissue and that silencing *METTL3* could lead to a decrease in proliferation, increased apoptosis, and thereby inhibit tumor growth in vivo and in vitro. Mechanistically, METTL3 promoted the expression of the oncoprotein hepatitis B virus X-interacting protein (HBXIP) which in turn, facilitated METTL3 expression by inhibiting miRNA *let-7g* which targets METTL3 for subsequent degradation (Cai et al. 2018). In addition, METTL3-mediated deposition of m^6^A at the *BCL-2* transcript increased its translation. *BCL-2* is one of the most important anti-apoptotic genes which facilitates the survival of tumor cells enhancing the breast cancer phenotype (Wang et al. 2020a). However, another study revealed that the expression of METTL3, together with METTL14 and WTAP, was significantly decreased in breast cancer (Wu et al. 2019). Therefore, according to this study, low m^6^A levels would promote breast tumorigenesis. Indeed, depletion of m^6^A at large internal exons results in prematurely polyadenylated transcripts, leading to nonfunctional tumor suppressor genes (Ni et al. 2018). In addition, the eraser FTO is up-regulated in breast cancer where it down-regulates the pro-apoptotic factor BNIP3 to mediate breast cancer proliferation, progression, and metastasis (Niu et al. 2019). Hypoxic environments and dysregulation of hypoxia-inducible factors (HIFs) lead to an adaptive response playing a central role in tumor progression and therapy resistance (Masson and Ratcliffe 2014). The expression of the eraser ALKBH5 was induced by hypoxia/HIF-dependent mechanisms, leading to decreased m^6^A levels that promoted the specification of breast cancer stem cells (CSC) (Zhang et al. 2016a). In addition, hypoxia also induced the expression of the oncogenic transcription factor ZNF217, promoting the breast CSC phenotype (Zhang et al. 2016b). The mouse orthologue ZFP217 has been shown to recruit the methyltransferase METTL3 into an inactive complex in embryonic stem cells (Aguilo et al. 2015), and hence, would cooperate with ALKBH5 in negatively regulating m^6^A levels and promoting breast tumorigenesis.

In summary, both high and deficient m^6^A levels might influence global expression programs that lead to malignant phenotypes, and the crosstalk among m^6^A readers, erasers and writers critically regulates the expression of key transcripts to maintain cellular homeostasis (Panneerdoss et al. 2018).

### Pseudouridine

Ψ (Fig. 4) was the first RNA modification identified in the early 1950s (Cohn and Volkin 1951; Davis and Allen 1957). It was originally described in tRNAs and has been detected in rRNAs, small nuclear RNAs (snRNAs), other ncRNAs, and mRNAs, representing the most abundant of all known RNA marks (Davis and Allen 1957; Reddy et al. 1972; Carlile et al. 2014; Lovejoy et al. 2014; Schwartz et al. 2014; Li et al. 2015; Adachi et al. 2019a,b). It consists of a posttranscriptional isomerization of uridine, resulting in the addition of an extra carbon–carbon bond between the base and the sugar, and a hydrogen bond donor (Cohn 1960; Charette and Gray 2000).

Ψ is an irreversible modification which can occur through two different mechanisms. The first is RNA-dependent, mediated by the H/ACA box small nucleolar ribonucleoproteins (snoRNPs) complex comprised of four conserved proteins, namely NHP2, GAR1, NOP10, and dyskerin (Yu et al. 2005; Hamma and Ferré-D'Amaré 2010; Ge and Yu 2013; Yu and Meier 2014; Adachi et al. 2019b). In contrast, the second is a highly conserved RNA-independent mechanism and involves different types of pseudouridine synthases (PUS enzymes) (Koonin 1996; Kaya and Ofengand 2003). These enzymes possess a conserved catalytic domain which enables them to recognize uridine substrates and convert them to Ψ (Hamma and Ferré-D'Amaré 2006; Rintala-Dempsey and Kothe 2017). Some members of the PUS enzyme family were identified as catalyzers of this dynamic, stress-induced modification on mRNA, specifically, PUS1, PUS7, and the mammalian homologs of yeast Pus4—TRUB1, TRUB2 (Carlile et al. 2014; Lovejoy et al. 2014; Schwartz et al. 2014; Li et al. 2015; Safra et al. 2017a; Carlile et al. 2019). For some of these enzymes, namely PUS7 and TRUB1, conserved consensus sequence motifs were detected in both yeast and human cells through an in vitro pseudouridylation assay (Carlile et al. 2019). Instead, only a weak, three-nucleotide sequence motif (HRU) was identified for PUS1, for which indeed a shared structure motif was detected (Carlile et al. 2019).

To date, only a few biological functions of Ψ on mRNAs have been identified (Adachi et al. 2019b; Borchardt et al. 2020). Previous studies have shown that pseudouridylation contributes to mRNA stabilization and the enhancement of translational capability in some mRNAs in vitro (Karikó et al. 2008; Anderson et al. 2010; Schwartz et al. 2014; Adachi et al. 2019b). In addition, artificial changes of U to Ψ in premature stop codons resulted in stop codon read-through both in vitro and in vivo, and in suppression of nonsense-mediated mRNA decay (Karijolich and Yu 2011; Adachi and Yu 2020). However, Ψ-containing mRNAs have also been shown to impede translation elongation and alter tRNA selection by the ribosome (Eyler et al. 2019). Thus, further work is needed to fully understand the role of pseudouridine in determining endogenous mRNAs fate.

The importance of Ψ in human pathology was highlighted by numerous studies associating its dysregulation in ncRNAs with diseases such as X-linked dyskeratosis congenita, cancer, diabetes, viral infections, heart defects, and inherited and mitochondrial disorders (Montanaro et al. 2006; Alter et al. 2009; Sieron et al. 2009; Liu et al. 2012; Fernandez-Garcia et al. 2013; Shaheen et al. 2016; Wang et al. 2016a; Zhao et al. 2016; Penzo et al. 2017; de Brouwer et al. 2018; Darvish et al. 2019; Shaheen et al. 2019; Watanabe et al. 2019; Nagasawa et al. 2020). Whether alteration of Ψ sites on mRNAs is also involved in these or other pathologies remains to be elucidated. Recent studies indicate, however, a potential connection. For instance, mutations in *PUS7* that segregate with intellectual disability and microcephaly lead to the abolishment of pseudouridylation not only in tRNAs but also in mRNAs (de Brouwer et al. 2018; Shaheen et al. 2019). Collectively, these results highlight the necessity for a deeper understanding of how mRNA pseudouridylation is related to human pathologies.

### Other mRNA modifications

#### N^6^, 2′-O-di-methyladenosine

Adjacent to the *N*^7^-methylguanosine (m^7^G) cap, the second nucleotide in many mRNAs can be methylated at the 2′-hydroxyl group; if the transcription start nucleoside is 2′-O-methyladenosine (A_m_), its N^6^ position can be further methylated to form N^6^, 2′-O-dimethyladenosine (m^6^A_m_) (Keith et al. 1978).

This modification stabilizes the mRNA by preventing DCP2-mediated decapping and microRNA-mediated mRNA degradation (Mauer et al. 2017). Unlike m^6^A, the biological function of m^6^A_m_ and its role in cellular homeostasis are still poorly understood.

m^6^A_m_ is a reversible modification catalyzed by the writer PCIF1/CAPAM (Akichika et al. 2019). *PCIF1/CAPAM* knockout cells are viable, but sensitive to oxidative stress (Akichika et al. 2019), a common adaptive advantage found in many types of cancer. Indeed, a genetic screen identified *PCIF1/CAPAM* as a putative tumor growth suppressor in bladder cancer (Hensel et al. 2015). m^6^A_m_ is also erased by the demethylase FTO (Mauer et al. 2017). Hence, whether a given phenotype resulting from the loss of *FTO* is due to defects in m^6^A or m^6^A_m_ metabolism is ambiguous and controversial: Whereas FTO has higher demethylase activity toward m^6^A_m_, the number of m^6^A sites in mRNA is at least 10-fold higher than the number of m^6^A_m_ sites. It has been proposed that FTO localization within the cellular compartments can vary between cell types and pathological states, being FTO-mediated demethylation of m^6^A and m^6^A_m_ prominent in the nucleus and in the cytoplasm, respectively (Wei et al. 2018a). In agreement with this observation, cytoplasmic FTO inhibits the CSC phenotype in colorectal cancer through its m^6^A_m_ demethylase activity. Hence, low FTO expression in patient-derived cell lines leads to increased m^6^A_m_ mRNA levels, resulting in enhanced tumorigenesis and chemoresistance (Relier et al. 2020).

#### N^1^-methyladenosine

Methylation at the N^1^ position in adenosine (m^1^A) confers a positive charge that can influence the local structure of the RNA or its interaction with RBPs. It can be found in several RNA species, including tRNA, rRNA and mRNA (Dominissini et al. 2016; Li et al. 2016b, 2017b; Safra et al. 2017b). In the particular case of this mark, the methyl group is added by distinct isoforms of the TRMT family of proteins, namely TRMT6/61A and TRMT10C, depending on the cytoplasmic or mitochondrial localization of the target mRNA (Safra et al. 2017b). The methyl group blocks the normal Watson–Crick base-pairing, resulting in erroneous incorporation and translation blocking. Early transcriptome-wide m^1^A-mapping studies suggested that thousands of transcripts could be decorated with the m^1^A mark (Dominissini et al. 2016; Li et al. 2016b). In addition, it was proposed that this modification correlated with higher translation efficiency when located in the 5′UTR of mRNA (Dominissini et al. 2016; Li et al. 2016b). However, a later m^1^A base-resolution mapping study revealed that m^1^A is not widespread on mRNAs and identified only ten and five cytosolic and mitochondrial m^1^A-modified transcripts, respectively (Safra et al. 2017b). The enzyme NADH dehydrogenase-5 (ND5) was among the m^1^A-marked mitochondrial mRNAs identified. *ND5* contains a single-nucleotide polymorphism that prevents the formation of m^1^A in the *ND5* mRNA. This mutation is linked to Leber's hereditary optic neuropathy, a hereditary disease leading to acute loss of central vision (Safra et al. 2017b).

The removal of m^1^A from mRNA is catalyzed by ALKBH3 (Li et al. 2016b). ALKBH3 is highly expressed in human tumors including prostate (Koike et al. 2012), non-small-cell lung (Tasaki et al. 2011), pancreatic (Yamato et al. 2012), and renal cell carcinoma (Hotta et al. 2015), and elevated ALKBH3 expression is associated with poor prognosis. However, whether high expression of ALKBH3 leads to aberrant m^1^A in cancer patients remains elusive. Notably, ALKBH3 also targets other substrates than m^1^A-marked RNA which also include abasic sites and methylated nucleosides of DNA (Westbye et al. 2008; Müller et al. 2010). The readers of the m^1^A modification include YTHDF1-3 and YTHDC1, although the downstream effect on the RNA fate and, therefore, disease outcome, remains to be elucidated (Dai et al. 2018b).

#### 5-methylcytosine

The methylation of carbon 5 in cytosine (m^5^C) was initially discovered in rRNAs and tRNAs. More recently, high-throughput techniques have revealed its presence in mRNAs, although its prevalence is limited (Yang et al. 2017).

In multicellular organisms, m^5^C is catalyzed by at least seven conserved RNA m^5^C methyltransferases of the NOL1/NOP2/SUN domain (NSUN) family of proteins (NSUN1–7) and DNMT2, being all of them specific for distinct RNA species. Although NSUN2 was originally described as a tRNA methyltransferase (Frye and Watt 2006; Goll et al. 2006; Tuorto et al. 2012), it can also methylate other ncRNA species (Khoddami and Cairns 2013) and mRNA (Yang et al. 2017). Several studies have shown that m^5^C sites are not randomly distributed—they are most abundant in proximity to the translation start codon, 3′UTRs, and near Argonaute-binding regions (Squires et al. 2012; Amort et al. 2017; Legrand et al. 2017; Yang et al. 2017). NSUN2-mediated m^5^C deposition influences mRNA translation (Tang et al. 2015; Bohnsack et al. 2019) nuclear-cytoplasmic shuttling (Yang et al. 2017), and mRNA stabilization (Chen et al. 2019). For instance, m^5^C deposition on the *HDGF* oncogene mRNA promotes its stabilization, therefore driving urothelial carcinoma of the bladder (Chen et al. 2019). *NSUN2* is a direct target of the oncogene Myc, and it is required for Myc-induced proliferation (Frye and Watt 2006). Consistently, NSUN2 is highly expressed in a range of tumors such as breast cancer, lymph-node metastases, and colorectal cancer (Frye et al. 2010; Okamoto et al. 2012; Yi et al. 2017). In gastric cancer, NSUN2 can suppress p57^Kip2^ and therefore promote tumor growth (Mei et al. 2020). In head and neck carcinoma, high NSUN2 expression adversely affects other tumor suppressors such as TP53, p16, and p27, increasing the risk of mortality (Lu et al. 2018). Depletion of *NSUN2* results in decreased growth of human squamous-cell-carcinoma xenografts, suggesting that NSUN2 could be potentially targeted for cancer therapy (Frye and Watt 2006). Importantly, whether the oncogenic phenotypes resulting from NSUN2 overexpression are specifically due to aberrant m^5^C deposition at mRNA, tRNA or both RNA species, needs to be further investigated.

#### 2′-O-methylation

2′-O-methylation (2′-O-Me) consists in the transfer of a methyl group at the 2′-hydroxyl of the ribose of all RNA species, predominantly of rRNA and tRNA (reviewed elsewhere in Ayadi et al. 2019; Dimitrova et al. 2019; Höfler and Carlomagno 2020).

Its deposition on mRNA was thought to occur only at the first 2 ribonucleotides (N1 and N2) within the 5′ cap structure (Langberg and Moss 1981; Inesta-Vaquera et al. 2018) being deposited solely by cap methyltransferase 1 (CMTR1) and 2 (CMTR2), respectively (Bélanger et al. 2010; Werner et al. 2011; Smietanski et al. 2014). Although, thousands of potential 2′-O-Me sites on coding regions of human mRNAs were identified by a detection technique known as Nm-seq (Dai et al. 2017), the same authors published a later corrigendum stating that this method was suitable to identify 2′-O-Me sites in rRNA but had led to false positives sites in mRNA due to mispriming contamination. According to the authors, a refined Nm-seq version was able to detect a similar distribution pattern as the original version, results confirmed by other techniques (Dai et al. 2018a). Nevertheless, further work to validate these newly identified sites is required.

In addition, the mechanism of 2′-O-Me deposition on coding transcripts is still to be revealed, but it might be guided by box C/D snoRNAs (SNORDs) as in the case of rRNA or directly mediated by single methyltransferases known to methylate other RNA species, such as FTSJ3 (Ge et al. 2010; Bartoli et al. 2018; Elliott et al. 2019).

The most studied function of 2′-O-Me on mRNA is recognition of self RNA by the immune system during viral infections (Daffis et al. 2010; Züst et al. 2011; Devarkar et al. 2016; Leung and Amarasinghe 2016; Encinar and Menendez 2020; Krafcikova et al. 2020; Morales et al. 2020). In addition, 2′-O-Me affects the stabilization of mRNA and translation, including the codon reading (Choi et al. 2018; Elliott et al. 2019). Moreover, 2′-O-Me at N^1^ prevents transcripts’ degradation by blocking decapping and exoribonuclease activities of DXO which degrades defectively capped pre-mRNAs (Jiao et al. 2013; Picard-Jean et al. 2018).

To date, no direct association has yet been established between 2′-O-Me on mRNA and human pathologies other than infections with viral agents, such as HIV or coronaviruses (Szretter et al. 2012; Ringeard et al. 2019; Krafcikova et al. 2020). However, some mediators of this mark, as for instance CMTR1, are involved in diverse pathologies, among which are asthma and cancer (Dahlin et al. 2015; Degryse et al. 2018; Du et al. 2018). In cancer, CMTR1 is overexpressed in T-cell acute lymphoblastic leukemia with *JAK3* mutations and undergoes gene rearrangements with *ALK*, producing a fusion protein promoting non-small-cell lung cancer development (Degryse et al. 2018; Du et al. 2018). The role of CMTR1 in these cancers is still to be determined, but its overexpression may cause increased stability or translation of specific oncogene transcripts, leading to tumor development. Furthermore, *CMTR2* was shown to be mutated in patients with lung adenocarcinomas (Campbell et al. 2016). Despite these observations, it has yet to be answered whether the disease phenotype derives from altered mRNA 2′-O-Me patterns induced by aberrant expression of such enzymes. Further work will be necessary to elucidate the mechanism of 2′-O-Me deposition and understand whether specific factors can act on all RNA species.

## DETECTION METHODS

**FIGURE 5.**
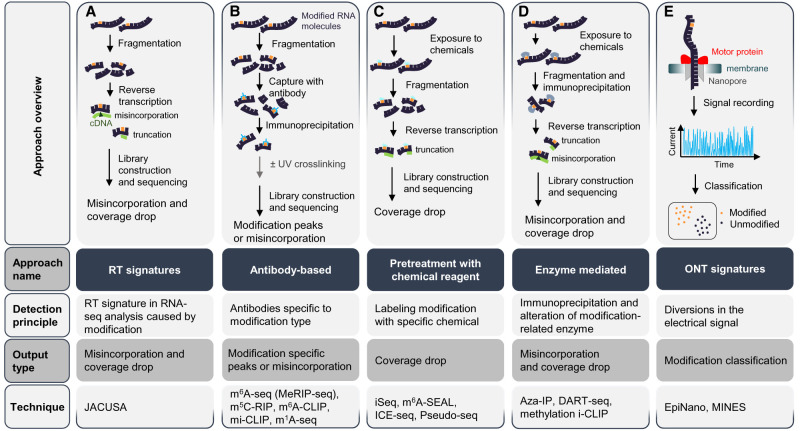
Approaches for the detection of RNA modifications. The figure presents the experimental approaches to the detection of RNA modifications, one per column and *left* to *right*: (*A*) Detection of nonrandom mismatch signatures (RT-signature), (*B*) capture with antibody, (*C*) pretreatment with chemical reagents, (*D*) capture with modification-related enzymes, and (*E*) detection of modification-specific signals using the Oxford Nanopore Technologies (ONT) platform (ONT-signature). Each column includes the approach schema, with rows *below* it indicating the approach name, its detection principle, output type, and techniques implementing that approach.

The efforts to detect, map, and quantify epitranscriptomic marks revealed many systematic properties of these marks, such as abundance, evolutionary conservation, reversibility, and biological function. Detection of A-to-I and C-to-U editing exploits a straightforward principle: Since the reverse transcriptase (RT) signatures can process both inosines (reading them as guanines) and uracils, so a discrepancy between RNA and DNA sequences can be detected by RT-PCR or high-throughput sequencing (Athanasiadis et al. 2004; Levanon et al. 2004; Rosenberg et al. 2011; Blanc et al. 2014; Oakes et al. 2017; Piechotta et al. 2017; Malik et al. 2021; Srinivasan et al. 2021). Moreover, bioinformatic approaches have been developed for improving quantitation (Fig. 5A; Piechotta et al. 2017; Cohen-Fultheim and Levanon 2021; Lerner et al. 2021; Lo Giudice et al. 2021), chemical modifications have been used to increase specificity (Fig. 5C; Cattenoz et al. 2013; Okada et al. 2019; Sakurai et al. 2021), and other approaches allow quantitation of specific editing in live cells (Garncarz et al. 2013; Chieca et al. 2021). Transcriptome-wide m^6^A-mapping methods, mostly represented by antibody-based techniques (Fig. 5B) such as methyl RNA immunoprecipitation followed by sequencing (MeRIP-seq or m^6^A-seq) and m^6^A individual-nucleotide-resolution crosslinking and immunoprecipitation (mi-CLIP or m^6^A-CLIP) (Dominissini et al. 2012; Meyer et al. 2012; Linder et al. 2015), have revealed a unique topology for this mark. However, these techniques can detect both m^6^A and m^6^A_m_ through the same antibody recognizing 6-methyladenine and hence, it is difficult to distinguish between the two marks within the mRNA 5′UTR (Fig. 5B; Linder et al. 2015; Hawley and Jaffrey 2019; McIntyre et al. 2020). To overcome potential biases of antibody-based techniques, pretreatment with chemical reagents (m^6^A-SEAL, Fig. 5C), or enzyme-mediated techniques (DART-seq, Fig. 5D; Liu et al. 2013; Meyer 2019; Vandivier et al. 2019; Wang et al. 2020b) have been developed, although they have not been widely used yet. Recently, nanopore-based sequencing developed by Oxford Nanopore Technologies (ONT), which allows the direct sequencing of native RNA, has also been used to investigate m^6^A (EpiNano and MINES, Fig. 5E). ONT signatures will advance our knowledge of m^6^A biology as this technology allows the novo identification of this and other marks at single-nucleotide resolution without RNA immunoprecipitation or pretreatment (Liu et al. 2019; Lorenz et al. 2020).

Ψ sites are detected by single-nucleotide resolution transcriptome-wide techniques based on combining high-throughput sequencing with chemical reagents pretreatment such as N-Cyclohexyl-N′-(2-morpholinoethyl) carbodiimide methyl-*p*-toluenesulfonate (CMC), a covalent adduct that blocks the RT activity (Fig. 5C; Carlile et al. 2014; Lovejoy et al. 2014; Schwartz et al. 2014; Li et al. 2015). Among these methods are pseudo-seq, Ψ-seq, PSI-seq, and CeU-seq (Li et al. 2016a; Penzo et al. 2017; Adachi et al. 2019a). Non-CMC based methods, such as RNA bisulfite sequencing (RBS-seq), have also been developed (Khoddami et al. 2019).

For the detection of the other mRNA modifications, transcriptome-wide mapping either by coupling an antibody-based approach to Dimroth rearrangement or by using an m^1^A-induced RT mismatch signature (namely m^1^A-seq, Fig. 5B), are used to detect the m^1^A mark (Dominissini et al. 2016; Li et al. 2016b). m^5^C can be determined by bisulfite sequencing (Fig. 5C) in which unmodified cytosines are converted to uracils after bisulfite treatment, whereas m^5^C sites are protected from deamination allowing the detection by high-throughput sequencing (Wei et al. 2018b). Additionally, m^5^C can be also detected by antibody-based techniques (Fig. 5B) such as m^5^C-seq and m^5^C-RIP or enzyme-mediated approaches (Fig. 5D) such as Aza-IP and methylation i-CLIP (Edelheit et al. 2013; Hussain et al. 2013; Khoddami and Cairns 2013). In conclusion, the detection of 2′-O-Me is currently performed by methods based on pretreatment with chemical reagents (Fig. 5C) such as RibOxi-seq and Nm-seq (Dai et al. 2017; Zhu et al. 2017; Motorin and Marchand 2018).

## PERSPECTIVES

RNA modifications have risen as major factors in posttranscriptional regulation of gene expression. However, the breadth of their action and its implications for cell physiology and pathology are still far from being sufficiently understood.

### Mechanism

The first aspect in need of further attention is the composition of the machinery behind these modifications. While writers and erasers were, in general, more thoroughly characterized, our knowledge of reader proteins is limited, and we are likely missing those proteins with secondary or moonlighting roles as modification readers. This knowledge will be instrumental in fully appreciating how these marks influence RNA fate. Secondly, while there is evidence supporting the possibility of a dynamic life cycle for several modifications, a neglected aspect is the magnitude of this dynamicity. Are RNA modifications continuously written and erased, or subject to less frequent cycles of deposition and degradation? Understanding this aspect could help elucidate their role in cellular processes requiring a fast response and in those with a slower unfolding.

### Interplay

Up to now, most of the epitranscriptomics literature focused on individual RNA marks, their physiological functions, and consequences of their dysregulation. Recently, new data suggested that different epitranscriptomic marks could coexist on the same transcript, and that a potentially widespread cooperative and competitive interplay could control the RNA fate, likely through RBPs (Dassi 2017). While the extent of this crosstalk is still unclear, several research groups described potential, mostly correlative, occurrences of such mechanisms (Li et al. 2017a; Dai et al. 2018b, 2020; Sokołowski et al. 2018; Wei et al. 2018a; Xiang et al. 2018; Huang et al. 2019; Seo and Kleiner 2020). For instance, both m^6^A and m^5^C methylation sites were found in a specific region of the *p21* mRNA. Furthermore, it has been shown that the m^6^A modification can facilitate m^5^C methylation and vice versa. This cooperation can synergistically enhance p21 translation in a model of oxidative stress-induced cellular senescence (Li et al. 2017a). This study also suggests that since NSUN2 (m^5^C) and METTL3/14 (m^6^A) methylate many coding and ncRNA species (Hussain et al. 2013; Liu et al. 2014; Kadumuri and Janga 2018), their interplay may act beyond *p21* (Li et al. 2017b) and affect several other transcripts. However, the prevalence of m^5^C in mRNAs is limited (Yang et al. 2017), and considerably lower than that of m^6^A. Thus, it is unclear whether this interplay can happen at a broader scale beyond *p21* mRNA, and further work will be necessary to understand its amplitude. Similarly, mRNAs encoding the four Yamanaka factors, exogenously modified with both Ψ and m^5^C, showed an increased efficiency in cellular reprogramming to a pluripotent state with respect to unmodified mRNAs (Warren et al. 2010). In both studies, the proximity of the modifications has been identified as the basis of their interplay. However, the molecular mechanisms behind this cooperation are still to be described. Also, a negative correlation between A-to-I editing and m^6^A methylation was observed (Xiang et al. 2018). In contrast to previous studies, this mutually exclusive interaction has been investigated and attributed to RNA structural features preventing ADAR1 binding, rather than direct competition (Xiang et al. 2018). Globally, while these studies highlight the co-occurrence of multiple marks on the same RNA molecules, the existence of direct cooperative and competitive mechanisms between those still needs to be demonstrated.

On the other end, RBPs controlling the life cycle of RNA modifications appear to interact with marks other than their canonical one. For instance, the YTHDF2 m^6^A reader may “integrate” epitranscriptomics marks by also reading m^1^A and m^5^C (Dai et al. 2018b, 2020; Lao and Barron 2019; Seo and Kleiner 2020). A conserved residue of YTHDF2 (Trp^432^) is required for the recognition of all three modifications, albeit the affinities for m^1^A and m^5^C are lower than those for m^6^A. This lower affinity, coupled to the scarcity of both marks in mRNAs, leaves the actual occurrence and phenotypic impact of this “integration” as a question still to be answered. If confirmed, also other YTH domain-family proteins could behave similarly (Dai et al. 2018b, 2020; Seo and Kleiner 2020), as might be expected given their observed redundancy (Zaccara and Jaffrey 2020). One may thus wonder how these mechanisms could induce or affect pathological states (Meier et al. 2016; Kadumuri and Janga 2018; Christofi and Zaravinos 2019; Huang et al. 2020b). Few studies have explored the relation between cancer and the crosstalk of different enzymes controlling the same modification (Panneerdoss et al. 2018), and how multiple modifications can concurrently control disease states has yet to be established. In Supplemental Table S1, we collect alterations of RNA modifications and editing in several diseases, obtained from the literature on this topic. As shown there, several tumor types are associated with the altered deposition of multiple modifications. This catalog could allow the identification of disorders associated with multiple RNA modifications and thus possibly affected by their interplay.

Overall, it appears that RNA marks may “talk” through direct cooperation or competition, and through the integration of multiple modifications by their reader proteins. Nevertheless, further work will be necessary to demonstrate the actual occurrence and elucidate the amplitude of this interplay, the complexity of the induced regulatory networks and its importance in shaping cell physiology. Is this behavior a form of “epitranscriptomic signaling,” allowing to coordinate the outcome of different pathways? And do changes in the RNA secondary structure interact to alter the transcript life cycle or are reader enzymes required for this crosstalk to modulate cell phenotypes? The answers could bring a new layer of complexity to epitranscriptomics, leading the field into uncharted avenues of even greater possibilities.

### Disease

Since RNA modifications represent such a basic layer in the biology of the cell, loss of these modifications is often fatal (Brusa et al. 1995; Wang et al. 2000; Geula et al. 2015) and might have profound effects on cell viability. This is probably the reason why only inactivating mutations in few genes associated with the epitranscriptome have so far been causatively linked to genetic diseases (Miyamura et al. 2003; Bykhovskaya et al. 2004; Rice et al. 2012). Yet, association of RNA modifications with the onset and progression of several human diseases is increasingly being uncovered. Particularly in cancer, several marks appear to play a key role in shaping the prognosis. However, the molecular mechanisms are still not fully understood. Are these modifications disease *drivers* or mere passengers? It is clear that in some cases alterations in the mediators of these RNA marks have direct effects on cellular tumor-promoting features (Frye et al. 2010; Qin et al. 2014). As such, alterations in these pathways could be selected in the cancer evolutionary process. On the other hand, a direct involvement in the onset of tumors has not been conclusively shown yet. Could these modifications be targeted to alter the course of the pathology? In Supplemental Table S1 we have highlighted the presence of opposite roles that can be played by RNA marks-regulating factors at an intra- and intertumor level. Can the affected pathways explain these disease-specific behaviors? Furthermore, tumors with potential alterations in multiple RNA marks have been identified. Can alterations to multiple enzymes controlling the life cycle of the different modifications be regarded as “double hits” leading to oncogenesis?

Answering these questions will greatly expand our knowledge on the role of epitranscriptomics in disease onset and progression, ultimately enabling us to design novel, highly specific therapeutic strategies against still incurable diseases.

## SUPPLEMENTAL MATERIAL

Supplemental material is available for this article.

## Supplementary Material

Supplemental Material
