# Supplementary material for: A mark of disease: how mRNA modifications shape genetic and acquired pathologies
Source: RNA. 2021 Apr;27(4):367–89. doi: 10.1261/rna.077271.120 (PMC7962492; doi:10.1261/rna.077271.120)
Supplement: Supplemental Material [file supp_27_4_367__index.html]

A mark of disease: how mRNA modifications shape genetic and acquired pathologies — A mark of disease: how mRNA modifications shape genetic and acquired pathologies — Supplemental Material 

# A mark of disease: how mRNA modifications shape genetic and acquired pathologies

## Supplemental Material

- Supplemental\_Table\_S1.xlsx
